# Rise in the number of notifications of Shiga toxin-producing *Escherichia coli* (STEC) infections probably linked to an increased use of multiplex PCR assays, Germany, 2023

**DOI:** 10.2807/1560-7917.ES.2025.30.48.2500268

**Published:** 2025-12-04

**Authors:** Tanja Jung-Sendzik, Mareike Wollenweber, Katja Hille, Lilas Mercuriali, Gerhard Falkenhorst

**Affiliations:** 1 Department of Infectious Disease Epidemiology, Robert Koch Institute, Berlin, Germany; 2 Postgraduate Training for Applied Epidemiology (PAE), Department of Infectious Disease Epidemiology, Robert Koch Institute, Berlin, Germany; 3 ECDC Fellowship Programme, Field Epidemiology path (EPIET), European Centre for Disease Prevention and Control (ECDC), Stockholm, Sweden; 4 Department of Infectious Disease Epidemiology and Surveillance, Public Health Agency of Lower Saxony, Hannover, Germany

**Keywords:** STEC, case notifications, multiplex PCR, laboratory survey

## Abstract

**BACKGROUND:**

Shiga toxin-producing *Escherichia coli* (STEC) can cause illnesses ranging from self-limiting diarrhoea to severe manifestations such as haemolytic-uraemic syndrome (HUS). In 2023, an increase in notified STEC cases was observed in the German federal state of Lower Saxony and nationwide.

**AIM:**

We aimed to investigate possible reasons for the observed increase.

**METHODS:**

We analysed data on notified STEC cases at federal and state level. All available STEC isolates from Lower Saxony from 2023 were whole genome sequenced. We sent a survey on detection and identification methods to 25 clinical microbiology laboratories in Lower Saxony.

**RESULTS:**

In 2023, a statistically significant increase in notified STEC cases in all ages was seen in Lower Saxony and nationwide when compared with case numbers in 2022 and the median of 2015–2019 (p < 0.01). The highest increase was observed in people aged 60–69 years: 110 cases were notified in Lower Saxony in 2023 (median 2015–2019: 26) and 471 cases nationwide (median 2015–2019: 182). No overall increase was seen in disease severity or in the number of HUS cases. No larger genetic clusters or outbreaks were identified in Lower Saxony. The survey among the 17 responding laboratories in Lower Saxony revealed an increased use of multiplex PCR assays for gastrointestinal pathogens, introduced mainly in 2023.

**CONCLUSION:**

The increase in notified STEC cases was probably associated with the implementation of multiplex PCR assays for the analysis of gastrointestinal specimens. Our findings highlight the need to monitor diagnostic practices when assessing and evaluating surveillance data.

**Key public health message**

**What did you want to address in this study and why?**

Infection with Shiga toxin-producing *E. coli* (STEC) may be asymptomatic or lead to a diarrhoeal disease with severe complications and death. In 2023, an increase in the number of cases with STEC was seen in the German federal state of Lower Saxony and nationwide. We aimed to investigate reasons for this increase.

**What have we learnt from this study?**

The increase in STEC notifications was probably linked to the introduction of multiplex PCR tests in routine laboratory diagnostics, rather than a true rise in infections. The increase was mainly seen in patients aged 60–69 years. We did not observe an increase in severe cases nor an indication of major outbreaks.

**What are the implications of your findings for public health?**

The introduction of multiplex PCR assays in routine diagnostic practices may have led to a more frequent detection of STEC, especially in older patients with gastrointestinal infections. Our findings highlight the importance of monitoring diagnostic practices when evaluating trends in surveillance data and planning health responses.

## Introduction

Shiga toxin-producing *Escherichia coli* (STEC) are Gram-negative bacteria that can colonise the human gastrointestinal tract and produce potent cytotoxins known as Shiga toxins (Stx1 and Stx2) with multiple subtypes. These toxins, particularly subtype Stx2a, can cause severe illnesses such as haemorrhagic colitis and haemolytic-uraemic syndrome (HUS), a potentially life-threatening condition characterised by acute kidney injury, microangiopathic haemolytic anaemia and thrombocytopenia [1]. Identification of STEC is usually done by the detection of Shiga toxins (e.g. by ELISA) or Shiga toxin genes (e.g. by PCR) [2]. Cultivation of the pathogen is recommended [2,3], but often not performed due to additional costs, time constraints and the lack of consequences for individual patient management.

Infectious disease surveillance in Germany is regulated by the Infection Protection Act (IfSG) of 2001 [4]. All laboratory-confirmed STEC cases, irrespective of the presence of symptoms, are to be notified to the local health departments [4]. From there, pseudonymised notifications are transmitted to the state health departments and further on to the National Public Health Institute, the Robert Koch Institute (RKI). Notifications of STEC include information on symptoms, hospitalisation status, possible place of infection and other variables, which are collected in the national database of notifiable diseases SurvNet@RKI [2,5]. In Germany, over 550 clinical microbiology laboratories perform microbiological diagnostics. Most of these laboratories are commercial, profit-oriented enterprises, accredited according to international standards [6,7]. Laboratories are reimbursed for PCR diagnostics of gastrointestinal pathogens since September 2022 within the statutory health insurance system [8].

According to the national guideline for acute infectious gastroenteritis in infants, children and adolescents, testing for STEC in children with gastrointestinal symptoms should be considered [9]. Independent of age, testing for STEC is recommended in severe cases with gastrointestinal symptoms and complications such as haemorrhagic colitis and HUS as well as in symptomatic people who work in the food sector or visit communal facilities [3,10]. Cases of enteropathogenic HUS are separately notifiable by treating physicians, based on clinical criteria.

We aimed to describe and analyse the observed STEC notification increase in 2023 and to identify possible reasons for the increase in Lower Saxony and Germany overall.

## Methods

### Data collection and analysis of STEC notifications

We extracted information of all notified STEC cases in Germany transmitted to the RKI between 2015 and 2023 from SurvNet@RKI [5].

In accordance with Section 11 (2) of the IfSG, the RKI has the task of developing surveillance case definitions for infectious diseases. Case definitions aim to ensure uniform criteria throughout Germany, thus contributing to standardised assessments and more reliable statistics. The national case definition for STEC is provided in Box.

Box **National case definition for infections of Shiga toxin-producing *Escherichia coli* (STEC), Germany**

**Clinical criteria:**

• Stomach pain or diarrhoea or vomiting or death

AND

**Laboratory confirmation:**

• Detection of Shiga toxin (e.g. by ELISA) from faecal specimen or stool culture

AND/OR

• Nucleic acid detection (e.g. PCR) of a Shiga toxin gene from faecal specimen or stool culture

OR

**Epidemiological confirmation:**

• At least one of the following, considering the incubation period (2–10 days):

o Epidemiological link to a laboratory-confirmed human case (human-to-human transmission) of STEC

OR

o Common source of exposure (e.g. bathing water, food, animal contact)

OR

o Bathing in a body of water, swimming pool or bathing water, detection of STEC laboratory-confirmed

OR

o Contact with an animal (e.g. at a petting zoo) or its faeces or consumption of its products (e.g. raw milk), detection of STEC laboratory-confirmed

OR

o Consumption of a foodstuff (including drinking water), detection of STEC laboratory-confirmed.

We compared STEC notification data from Lower Saxony and nationwide from 2023 with data from 2022 and with the median of the five pre-COVID-19 pandemic years 2015–2019. We assessed changes over time in the following variables: case numbers, age and sex distribution, proportions of asymptomatic cases, and HUS case numbers. We excluded the pandemic years 2020 and 2021 from our analyses due to a drastic decrease in notifications for most infectious diseases [11]. We contacted local health authorities regarding results of their case investigations.

Increases in different age groups in 2023 were compared with the reference periods 2022 and 2015–2019 by two-sample Poisson test. Hospitalisation rates due to STEC among all STEC cases were analysed as a proxy for disease severity by chi-square test to assess whether 2023 rates differed significantly from those in 2018–2019 and 2022. The years 2018–2019 and 2022 were selected as reference periods based on stable hospitalisation rates. We considered p values < 0.05 statistically significant. Analyses were performed in Excel version 2019, R version 4.3.0 (https://www.r-project.org), and Stata version 19.00.20251006 (https://www.stata.com). Maps were generated in RegioGraph, version 2018 (https://regiograph.gfk.com).

### Laboratory survey

To investigate whether the observed increase in STEC notifications was associated with changes in laboratory diagnostic routines and/or changes in reimbursement procedures, we conducted a laboratory survey in Lower Saxony, using the online-platform LamaPoll (https://www.lamapoll.de). The survey contained eight questions on methods and assays used for STEC diagnostics, the time of implementation of these methods and changes in diagnostic routines. The survey questionnaire is available in Supplement S1.

An invitation to the survey was sent via email to 25 clinical microbiology laboratories which participate in the Lower Saxony antimicrobial resistance monitoring network, as well as in the national network of accredited medical laboratories [12,13]. These participants represented most of the accredited clinical microbiology laboratories within the federal state. Laboratories were evenly distributed across Lower Saxony. Some were located in the neighbouring city states Bremen and Hamburg that also processed specimens from patients residing in Lower Saxony. The survey was carried out between 12 December 2023 and 8 February 2024. All participating laboratories provided their informed consent before participation.

### Genomic analyses

All available STEC cases isolates (n = 120) from Lower Saxony in 2023 were characterised by whole genome sequencing (WGS) by short-read paired-end sequencing performed using MiSeq (2 × 300 bp), HiSeq 1500 (2 × 250 bp) and NextSeq 1000/2000 (2 × 150 bp) instruments (Illumina, San Diego, the United States (US)). Whole genome sequencing was performed at the National Reference Centre (NRC) for salmonellosis and other enteric pathogens at the RKI, or at the federal state laboratory of Lower Saxony. Genetic relationships were assessed by core genome multilocus sequence typing (cgMLST) according to the Enterobase scheme [14]. A genetic cluster was defined as isolates displaying < 5 allele differences in cgMLST.

## Results

### Spatial distribution

**Figure 1:**
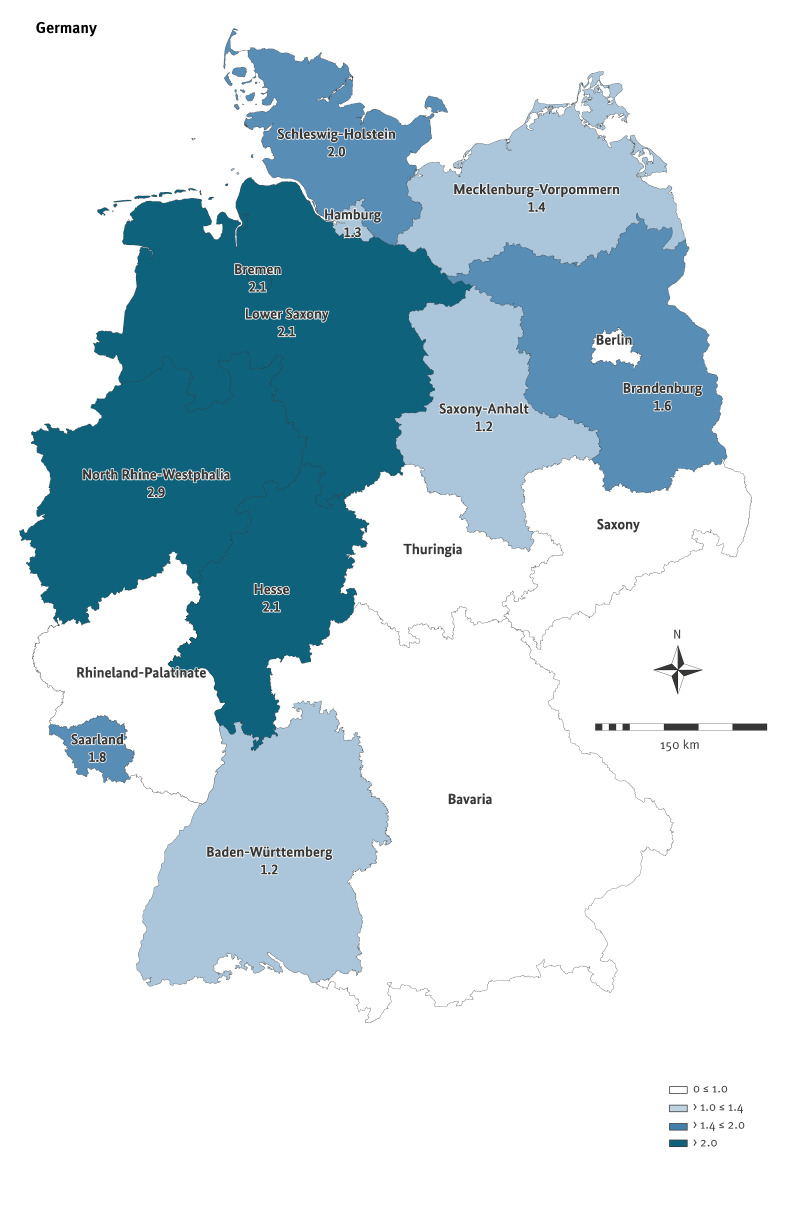
Fold changes to notifications of cases with Shiga toxin-producing *Escherichia coli* (STEC) infection in 2023 (n = 4,458 cases) compared with the median of 2015–2019 (n = 2,657 cases), by federal state, Germany

**Figure 2:**
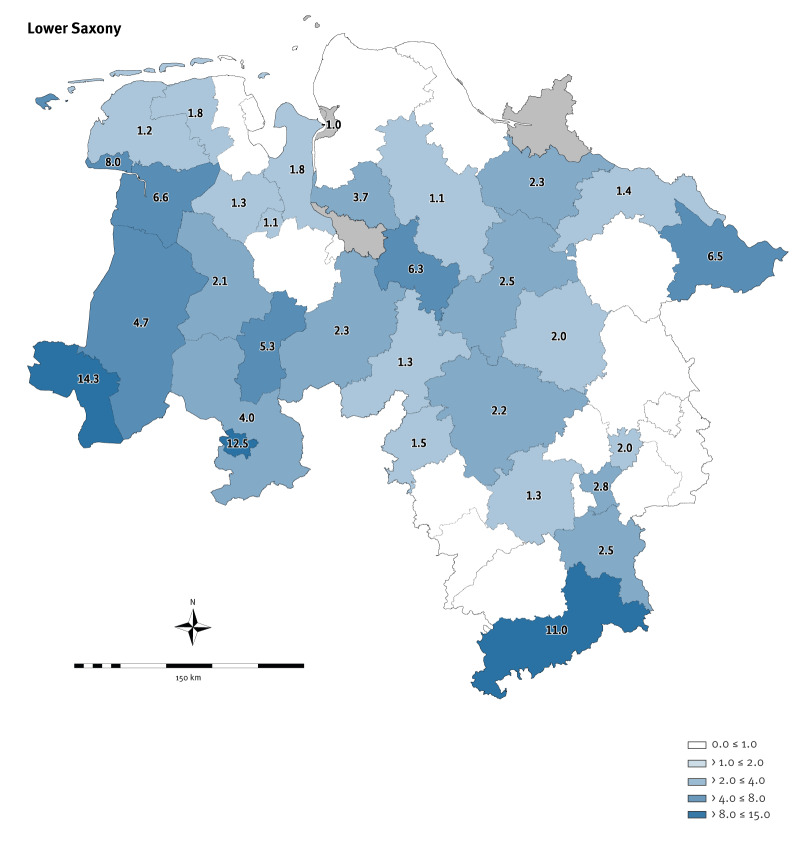
Fold changes to notifications of cases with Shiga toxin-producing *Escherichia coli* (STEC) infection in 2023 (n = 762 cases) compared with the median of 2015–2019 (n = 363 cases), by district, Lower Saxony, Germany

In Lower Saxony, 762 STEC cases were notified in 2023, equalling a 2.3-fold increase compared with 2022 (337 cases), and a 2.1-fold increase compared with the median of 2015–2019 (363 cases). Nationwide, a similar finding was observed with 4,458 notified STEC cases in 2023, equalling a 1.8-fold increase compared with 2022 (2,441 cases), and a 1.7-fold increase compared with the median of 2015–2019 (2,657 cases). These increases were statistically significant (p < 0.01). The increase in STEC notifications was not uniformly distributed across the 16 German federal states (Figure 1). Highest increases were seen in Lower Saxony (population: 8.0 million) and the neighbouring North Rhine-Westphalia (population: 18.0 million) which together account for 31% of the German population of 83.5 million [15]. Case numbers from these two federal states (2,237), constituted 50% of all STEC notifications (4,458) in Germany in 2023. Within Lower Saxony, the rise differed between districts (Figure 2). In other states, such as Baden-Wuerttemberg, Hamburg, Rhineland-Palatinate, Saxony and Saxony-Anhalt, no or only a slight increase was seen compared with the median 2015–2019, whereas in Bavaria, Berlin and Thuringia, case numbers slightly decreased (Figure 1). Detailed notification numbers for each federal state between 2015 and 2023 can be found in Supplementary Table S1.

The colouring of the increase in notifications is explained in the box in the lower right corner of the map.

The colouring of the increase in notifications is explained in the box in the lower right corner of the map. The city states of Bremen and Hamburg are depicted in grey.

**Figure 3:**
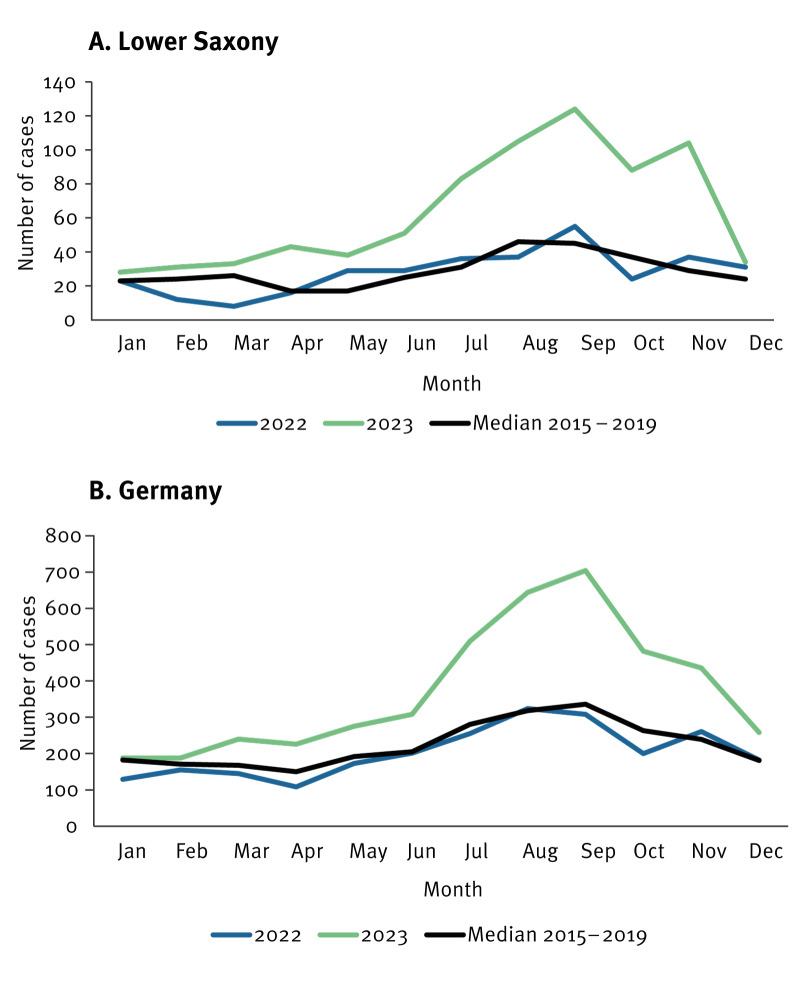
Number of notified cases with Shiga toxin-producing *Escherichia coli* (STEC) infection in 2023 compared with 2022 and the median of 2015–2019, by month, Lower Saxony (A) and Germany (B)

While STEC notifications in Lower Saxony and nationwide in 2022 were at the same level as the median of 2015–2019, they were consistently higher over the course of 2023 (p < 0.01), with the most noticeable increase starting in June, reaching peaks by August–September (Figure 3).

Panel A. Number of notified cases: 2023 (n = 762); 2022 (n = 337); median of 2015–2019 (n = 363).

Panel B. Number of notified cases: 2023 (n = 4,558); 2022 (n = 2,441); median of 2015–2019 (n = 2,657).

Case interviews performed by local health authorities in Lower Saxony did not reveal any shared exposures of the STEC cases.

### Age and sex distribution

**Figure 4:**
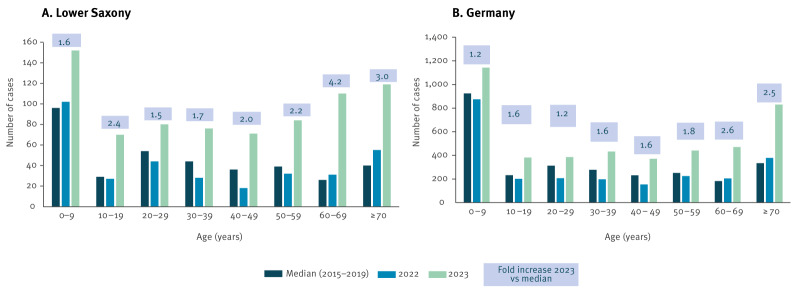
Age distribution of notified cases with Shiga toxin-producing *Escherichia coli* (STEC) infection, Lower Saxony (A) and Germany (B), 2015–2023

Notifications of STEC increased in all age groups in 2023 compared with 2022 and the medians of 2015–2019 (p < 0.01). In Lower Saxony, compared with the 2015–2019 medians, these increases ranged from 1.5-fold in age group 20–29 years (2023: 80 cases; median 2015–2019: 54 cases), up to 4.2-fold in those aged 60–69 years (2023: 110 cases; median 2015–2019: 26 cases) (Figure 4A). The median age of cases was 40 years in 2023, 29 years in 2022 and 31 years in 2015–2019. Nationwide, the increases in 2023, when compared with the 2015–2019 medians, ranged from 1.2-fold in persons aged 0–9 years (2023: 1,143 cases; median 2015–2019: 924 cases) and 20–29 years (2023: 385 cases; median 2015–2019: 313 cases) up to 2.6-fold in those aged 60–69 years (2023: 471 cases; median 2015–2019: 182 cases) (Figure 4B). The median age of STEC cases nationwide was 36 years in 2023, 26 years in 2022 and 27 years in 2015–2019.

Panel A. Number of notified cases: 2023 (n = 762); 2022 (n = 337); median of 2015–2019 (n = 363).

Panel B. Number of notified cases: 2023 (n = 4,453); 2022 (n = 2,440); median of 2015–2019 (n = 2,655). In all age groups, the increases in 2023 were statistically significant compared with 2022 and 2015–2019 (p < 0.01). Due to missing information on age groups in the surveillance system, there are five fewer cases in the total age numbers (n = 4,453) compared with the total STEC notification numbers (n = 4,558).

The proportion of female cases among notified cases in Lower Saxony was similar in 2023 (52.2%; 398/762) and in 2015–2019 (51.2%; 186/363) and slightly higher in 2022 (55.5%; 187/337). At the national level, the proportion of female cases was 53.2% (2,373/4,458) in 2023, 51.1% (1,359/2,658) in 2015–2019, and 54.1% (1,320/2,441) in 2022. Detailed results are available in Supplementary Table S2.

### Clinical presentation

The number of STEC cases reported to be asymptomatic or without information on the presence of symptoms differed between the years, with a smaller proportion of asymptomatic cases in Lower Saxony in 2023 (21%; 160/762) compared with the median in 2015–2019 (32%; 117/363). However, in age groups 0–9 and 60–69 years, the proportions in 2023 (0–9 years: 22%; 33/152 and 60-69: 22%; 24/110) were comparable to the median (21% for each of the two age groups). Nationwide, similar findings were observed and are presented in Supplementary Figure S1.

### Hospitalisation

**Figure 5:**
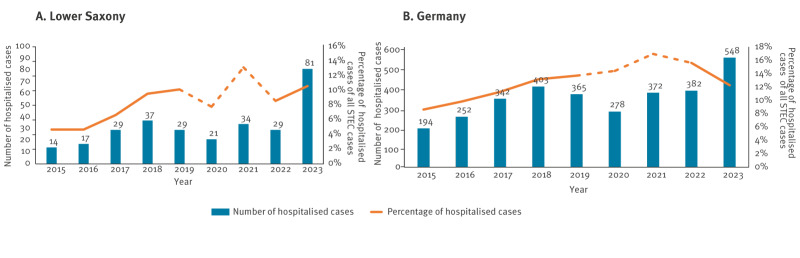
Number of cases hospitalised due to Shiga toxin-producing *Escherichia coli* (STEC) infection, and proportions of cases hospitalised due to STEC among all notified STEC cases, Lower Saxony (A) and Germany (B), 2015–2023

Hospitalisations due to STEC increased in Lower Saxony from 29 cases in 2022 and 29 median cases in 2015–2019 to 81 cases in 2023. Nationwide, hospitalisations due to STEC increased from 382 cases in 2022 and 341 median cases in 2015–2019 to 548 in 2023 (Figure 5).

Proportions during the COVID-19-pandemic years are shown as dotted line.

Panel A. Number of notified STEC cases (n1) and cases hospitalised due to STEC (n2) : 2015 (n1 = 298; n2 = 14); 2016 (n1 = 363; n2 = 17); 2017 (n1 = 435; n2 = 29); 2018 (n1 = 386; n2 = 37); 2019 (n1 = 285; n2 = 29); 2020 (n1 = 269; n2 = 21); 2021 (n1 = 258; n2 = 34); 2022 (n1 = 337; n2 = 29); 2023 (n1 = 762; n2 = 81); median of 2015–2019 (n1 = 363; n2 = 29).

Panel B. Number of notified STEC cases (n1) and cases hospitalised due to STEC (n2): 2015 (n1 = 2,245; n2 = 194); 2016 (n1 = 2,559; n2 = 252); 2017 (n1 = 3,029; n2 = 342); 2018 (n1 = 3,055; n2 = 403); 2019 (n1 = 2,658; n2 = 365); 2020 (n1 = 1,924; n2 = 278); 2021 (n1 = 2,189; n2 = 372); 2022 (n1 = 2,441; n2 = 382); 2023 (n1 = 4,458; n2 = 548); median of 2015–2019 (n1 = 2,658; n2 = 342).

We considered proportions of cases hospitalised due to STEC of all notified STEC cases as a proxy for disease severity. The years 2018, 2019 and 2022 were selected as reference periods based on stable hospitalisation proportions. In Lower Saxony, the proportion of cases hospitalised due to STEC did not differ between these years (p > 0.05). Nationwide, the proportion of hospitalised cases in 2023 (548/4,458; 12%) was significantly lower than in 2022 (382/2,441; 16%; p < 0.05), but not lower than in 2018 (403/3,055; 13%) or 2019 (365/2,658 cases; 14%; p > 0.05) (Figure 5).

### Cases with haemolytic-uraemic syndrome (HUS)

**Figure 6:**
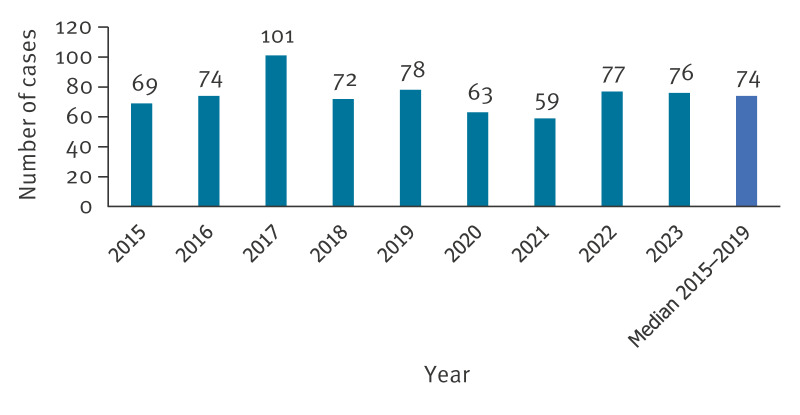
Number of notified Shiga toxin-producing *Escherichia coli* (STEC) infection cases with haemolytic-uraemic syndrome, Germany, 2015–2023 (n = 669)

Contrary to the rise in STEC cases in 2023, we did not see a nationwide increase in HUS cases from 2022 to 2023 (77 vs 76 cases) or from the median of 2015–2019 (74 cases). A peak of 101 cases was observed in 2017 due to an outbreak of sorbitol-fermenting STEC O157 [16] (Figure 6). In Lower Saxony, 9 HUS cases were notified in 2023, six in 2022 and a median of 12 cases in 2015–2019.

Number of notified cases with haemolytic-uraemic syndrome: 2015 (n = 59); 2016 (n = 74); 2017 (n = 101); 2018 (n = 72); 2019 (n = 78); 2020 (n = 63); 2021 (n = 59); 2022 (n = 77); 2023 (n = 76); median of 2015–2019 (n = 74).

### Laboratory survey

Of the 25 invited clinical microbiology laboratories, 17 fully completed the questionnaire (response rate 68%). Multiple entries on procedures were possible.

Ten laboratories reported the use of multiplex PCR panels for gastrointestinal pathogens for STEC detection (Table). Six laboratories had introduced multiplex PCR in 2023. Five laboratories used Seegene Allplex GI-EB panel (Seegene, Seoul, South Korea) only, two laboratories used R-biopharm RIDAGENE EHEC/EPEC (R-biopharm, Darmstadt, Germany) and two laboratories used both panels. One laboratory applied the Luminex NxTAG Gastrointestinal Pathogen Panel (Luminex, Austin, the US).

**Table:** Laboratory methods used for detection and identification of Shiga toxin-producing *Escherichia coli* in clinical microbiological laboratories, Lower Saxony, Germany, 2023–2024 (n = 17)

Method	Laboratories (n)
Multiplex PCR panels for gastrointestinal illnesses	10
PCR/RT-PCR (*stx1*, *stx2*, *eaeA* genes) directly from faecal specimens	6
PCR/RT-PCR (*stx1, stx2, eaeA* genes) from colony washing	1
ELISA/EIA from enrichment broth	4
Faecal culture	2
Serotyping	1

*eae*: gene coding for intimin; *stx*: Shiga toxin gene.

Information collected between 12 December 2023 and 8 February 2024; 17 of 25 invited laboratories responded. Multiple entries were possible.

Six laboratories used PCR assays for detection of STEC virulence genes (*stx1*, *stx2*, *eaeA*) directly from the stool specimens. Four of these laboratories had implemented this method in 2023. Two of the 17 laboratories cultured faecal samples.

Fifteen laboratories had not observed an increase in specific requests for STEC diagnostics. Laboratories noted that the implementation of multiplex PCRs in combination with a change in reimbursement in 2022 for PCR diagnostics may have led to an increase in STEC diagnoses.

### Sequencing results

A total of 120 STEC isolates from notified cases across Lower Saxony were sequenced (WGS). Most isolates were genetically heterogenous, only six clusters consisting of two cases, each with < 5 allele differences were identified.

## Discussion

In 2023, Lower Saxony observed a 2.3-fold increase in STEC notifications compared with 2022, and 2.1-fold compared with the median of 2015–2019. Nationwide, a similar finding was observed with a 1.8-fold increase compared with 2022, and a 1.7-fold increase compared with the median of 2015–2019. We aimed to investigate whether these increases in notifications were due to a true rise in infections or caused by changes in diagnostic procedures.

The nationwide case increase varied across the 16 German federal states (median 2015–2019 to 2023: 0.7–2.9-fold; 2022 to 2023: 1.1–2.3-fold). Highest increases were observed in Lower Saxony and North Rhine-Westphalia which together constituted for 50% of all STEC notifications in Germany in 2023. Case numbers increased in all age groups, both in Lower Saxony and nationwide when compared with the reference periods 2022 and 2015–2019. The strongest increase was observed in individuals aged 60–69 years. We did not detect any major changes in the sex distribution.

Nationwide, the proportion of hospitalisations due to STEC among all notified STEC cases was significantly lower in 2023 than in 2022. This could indicate a more frequent detection of non-severe STEC infections, not leading to hospitalisations. In Lower Saxony, however, we did not observe any statistically significant changes in hospitalisation proportions.

Whole genome sequencing of all available STEC isolates from 2023 from cases in Lower Saxony showed a wide genetic variety with no indication of clusters of more than two cases. Moreover, case interviews which were conducted by local health authorities as part of their routine case investigations, did not find evidence of shared exposures in Lower Saxony. Thus, a common source of infection was an unlikely explanation for the increase in case notifications.

To identify changes in diagnostic procedures that may explain the increase in STEC notifications in Lower Saxony, we performed a laboratory survey. Ten of 17 clinical microbiology laboratories performed multiplex PCR assays for testing of patients with gastroenteritis, and six of those had introduced these assays in 2023. Since September 2022, laboratories in Germany are officially reimbursed for nucleic acid detection of bacteria causing gastroenteritis, including STEC [8], which may have triggered the implementation of multiplex PCR panels. The use of multiplex PCR may also explain the higher increase of notifications in adults compared with children, since adults with gastrointestinal symptoms were previously not routinely tested for STEC, and thus would not have been diagnosed as STEC cases in the past.

Similar increases in STEC notifications after implementation of multiplex PCR have been reported from other countries such as Denmark and Norway, the US and Australia [17-22]. Norway observed an increase in low virulent STEC strains, without an increase in HUS cases [17] and Denmark an increase in STEC cases in older adults without an increase in disease severity [18,23].

The German national guideline for gastrointestinal infections [3] recommends testing for gastrointestinal infections only if the result is expected to have medical, organisational or notification consequences. Primary testing should include *Campylobacter, Salmonella*, rotavirus and norovirus. Infections of STEC are more frequent in the paediatric population, and children aged < 5 years are at increased risk [24]. Testing for STEC is therefore routinely recommended in symptomatic children and in severe cases with bloody diarrhoea or in symptomatic people who work in the food sector or visit communal facilities [3,9,10]. In case of a laboratory-confirmed STEC infection, children are not allowed to attend daycare or school; food industry workers are banned from work as long as they shed STEC.

Isolates are still essential for monitoring circulating STEC serotypes, cluster detection using WGS, epidemiological investigations and antimicrobial susceptibility testing [20,25,26]. The RKI and the Guideline for Gastrointestinal Infections recommend laboratories to perform bacterial culture on PCR-positive specimens [2,3], so-called reflex cultures. In our survey, 10 of the 17 participating laboratories reported the use of multiplex PCR panels, however, none of them reported to perform additional reflex cultures.

Detection of STEC by culture methods is challenging and labour-intensive due to the need for special selective media. The use of multiplex PCR allows shorter turnaround times, simplified workflows [20,21], automation of processes without deep technical knowledge and cost reductions [27]. Timely diagnosis can improve patient care and assist in infection control [20,26,28]. A drawback of PCR is that it detects the genetic material of pathogens regardless of their viability or ability to cause symptoms, which is one of the reasons the national guideline cautions against a universal use of multiplex PCRs. Instructions of commercial multiplex PCR assays stipulate that up to 45 amplification cycles should be run for nucleic acid detection. The German NRC for salmonellosis and other enteric pathogens observed that STEC cannot usually be detected by culture methods from stool specimens that were positive by PCR with quantification cycle (Cq) values > 32 (A Fruth, NRC, June 2024, personal communication). Therefore, it is questionable if positive PCR results with Cq values > 32 indicate true STEC infections or persisting infectiousness when used in follow-up stool examinations of cases. Without culture confirmation, PCR results with high Cq values may lead to unnecessary exclusions of children from daycare facilities or schools, as well as staff members, often with substantial social and economic consequences [17,21,29,30]. Moreover, the commercial multiplex PCR panels currently available in Germany do not differentiate between *stx1* and *stx2*, possibly further contributing to unnecessary exclusions, as negative follow-up stool examinations would not be required to lift exclusions for cases with *stx2*-negative STEC isolates [30]. The differentiation between *stx1* and *stx2* would enable a more targeted patient management and follow-up, as the detection of less virulent strains could lead to fewer and/or shorter exclusions from daycares and workplaces.

Several pathogens can be detected in a single stool specimen from a patient with gastroenteritis using multiplex PCR. Studies of multiplex PCR performed in children with gastroenteritis indicated that around 20–50% of positive stool specimens were positive for more than one pathogen [21,27,28,31] but the symptom-causing pathogen was not always identified. Little is known, however, whether this also applies to adults. Contrary to the increase in STEC, notifications of other enteropathogenic bacteria, such as *Campylobacter, Yersinia* or *Salmonella,* did not increase in Germany during the study period [32].

Our analyses relied on case characteristics, such as presence of symptoms and hospitalisation status, as reported by local health authorities in the notification system. While there may be reporting errors, we have no reason to believe that the accuracy of the notification data has considerably changed over the study period. As we did not investigate other German federal states, we do not know reasons for the varying notification rates. Additional laboratory surveys may need to be carried out in other federal states to further investigate the increase in notifications. Moreover, a possible change in specimen submission behaviour by physicians could be explored.

## Conclusion

We observed a marked increase in STEC notifications in 2023 in Lower Saxony and Germany nationwide, compared with 2022 and the median of 2015–2019. We consider the implementation of multiplex PCR panels for gastrointestinal pathogens in routine diagnostics to be the most plausible explanation for the observed increase. Our findings underscore the importance of monitoring diagnostic practices when evaluating trends in surveillance data.

## Data Availability

Query of notification data in accordance with the Infection Protection Act (IfSG) via SurvNet@RKI. A limited dataset without information on individual STEC and HUS cases can be downloaded from: https://survstat.rki.de/default.aspx

## Disclaimer

Tanja Jung-Sendzik and Mareike Wollenweber were fellows of the German Postgraduate Training in Applied Epidemiology (PAE) at RKI, associated with the ECDC Fellowship Programme. Lilas Mercuriali was fellow of the of the ECDC Fellowship Programme, supported financially by the European Centre for Disease Prevention and Control (ECDC). The views and opinions expressed herein do not state or reflect those of ECDC. ECDC is not responsible for the data and information collation and analysis and cannot be held liable for conclusions or opinions drawn.

## Supplementary Data

469000 Supplementary Material
