# Supplementary material for: Rise in the number of notifications of Shiga toxin-producing Escherichia coli (STEC) infections probably linked to an increased use of multiplex PCR assays, Germany, 2023
Source: Euro Surveill. 2025 Dec 4;30(48):2500268. doi: 10.2807/1560-7917.ES.2025.30.48.2500268 (PMC12680918; doi:10.2807/1560-7917.ES.2025.30.48.2500268)
Supplement: Supplementary Material [file 25-00268_JUNG-SENDZIK_Supplement.pdf]

Supplements:
This supplementary material is hosted by Eurosurveillance as supporting information alongside the article
“Rise in the number of Escherichia coli (STEC) infections probably linked to an increased use of multiplex-PCR assays, Germany, 2023”,
on behalf of the authors, who remain responsible for the accuracy and appropriateness of the content.
The same standards for ethics, copyright, attributions and permissions as for the article apply.
Supplements are not edited by Eurosurveillance and the journal is not responsible for the maintenance of any links or email addresses provided therein.

Supplement S1 - Questionnaire

Fragebogenentwurf zur Laborbefragung in Zusammenhang mit dem Fallzahlanstieg von EHEC 2023 – Online Befragung via LamaPoll

- Einleitungstext –

Transparenz- und Informationspflichten nach der Datenschutz-Grundverordnung/Information zur Befragung im Rahmen einer Ausbruchsuntersuchung

1 INFORMATION ZUR BEFRAGUNG

1.1 ***Ich habe die 'Information zur Befragung im Rahmen einer Ausbruchsuntersuchung' gelesen und verstanden und ich stimme der Teilnahme an der Befragung freiwillig zu.***

☐ Ja

☐ Nein

2 ABGEKÜRZTE BEFRAGUNG

2.1 *Sie können nur an der Umfrage teilnehmen, wenn Sie die "Information zur Befragung im Rahmen einer Ausbruchsuntersuchung" gelesen haben und der Teilnahme zustimmen. Bitte gehen Sie zurück und lesen die Information aufmerksam durch und erklären Sie Ihre Bereitschaft zur Teilnahme über die Schaltfläche "Ja". Wenn Sie nicht an der Umfrage teilnehmen möchten, klicken Sie bitte auf weiter. Sie werden dann zum Ende der Umfrage geleitet.*

NGA

3 IDENTIFIZIERUNG

3.1 Bitte geben Sie den Namen Ihres Labors an

Name: Freitext

3.2 In welchem Landkreis/Stadtkreis befindet sich das Labor

☐ Ammerland

☐ Aurich

☐ Braunschweig

☐ Bremen

☐ Celle

☐ Cloppenburg

☐ Cuxhaven

☐ Delmenhorst

☐ Diepholz

☐ Emden

☐ Emsland

☐ Friesland

☐ Gifhorn

☐ Goslar

☐ Göttingen

☐ Grafschaft Bentheim

☐ Hameln-Pyrmont

☐ Hannover (Region)

☐ Harburg

☐ Heidekreis

☐ Helmstedt

☐ Herzogtum Lauenburg

☐ Hildesheim

☐ Holzminden

☐ Leer

☐ Lüchow-Dannenberg

☐ Lüneburg

☐ Nienburg/Weser

NLGA

☐ Northeim

☐ LK Oldenburg

☐ SK Oldenburg (Oldb)

☐ LK Osnabrück

☐ SK Osnabrück

☐ Osterholz

☐ Peine

☐ Rotenburg (Wümme)

☐ Salzgitter

☐ Schaumburg

☐ Stade

☐ Uelzen

☐ Vechta

☐ Verden

☐ Wesermarsch

☐ Wilhelmshaven

☐ Wittmund

☐ Wolfenbüttel

☐ Wolfsburg

☐ Andere: _______________

NLGA

4 METHODIK

4.1 Welche Methode/n verwenden Sie standardmäßig für die EHEC/HUS Diagnostik?

☐Stuhlkultur

☐Multiplex-PCR für Gastrointestinale Erkrankungen, direkt aus dem Stuhl

☐PCR/RT-PCR (stx1, stx2, eae Gene) direkt aus dem Stuhl

☐PCR/RT-PCR (stx1, stx2, eae Gene) aus Kolonieabschwemmung

☐PCR/RT-PCR (stx1,stx2,eae) aus Anreicherungsbouillon

☐ELISA/EIA aus der E.-coli-Kultur

☐ELISA/EIA aus Anreicherungsbouillon

☐ELISA/EIA direkt aus dem Stuhl (nicht empfohlen)

☐Serotypisierung

☐Genomsequenzierung

☐LPS-Antikörper gegen E. coli O157 (HUS)

☐Sonstiges: _____________

4.2 Seit wann benutzen Sie Multiplex PCR für den Nachweis von gastrointestinalen Erkrankungen?

Datum: _____________

4.3 Seit wann ungefähr benutzen Sie PCR/RT-PCR direkt aus dem Stuhl für den Nachweis von EHEC?

Datum: _____________

4.4 Seit wann ungefähr benutzen Sie PCR/RT-PCR aus Kolonieabschwemmung für den Nachweis von EHEC?

Datum: _____________

4.5 Seit wann ungefähr benutzen Sie PCR/RT-PCR aus Anreicherungsbouillon für den Nachweis von EHEC?

Datum: _____________

4.6 Welches Multiplex PCR-Kit verwenden Sie? (von welchem Hersteller?)

Freitext: ________________

NLGA

4.7 Bei welchem CT-Wert setzen sie den Cut-off für ein positives Ergebnis an?

CT-Wert: ________________

4.8 Wurden Ihre Standard-Diagnostikverfahren für EHEC seit 2020 geändert?

☐ Ja (bitte erläutern, was? wann?) ______________________

☐ Nein

☐ keine Angabe

4.9 Melden Sie Ihre EHEC/HUS Fälle via DEMIS?

☐ Ja (bitte erläutern: seit wann?) ______________________

☐ Nein

☐ keine Angabe

4.10 Haben Sie eine Zunahme der Anforderungen zur EHEC/HUS Labordiagnostik festgestellt?

☐ Ja

☐ Nein

☐ keine Angabe

4.11 Haben Sie eine Erklärung oder Idee für die erhöhte Zahl der gemeldeten EHEC-Fälle im Jahr 2023? Falls ja, bitte erläutern.

Freitext: ____

4.12 Dürfen wir Sie Kontaktieren, falls wir noch Fragen zu Ihren Angaben haben?

☐ Ja

☐ Nein

Falls „Ja“ angekreuzt:

Name: ____

E-Mail: ____

Telefon: ____

Herzlichen Dank für Ihre Mitarbeit!

NLGA

Supplement S 2:

Table S1: Absolute numbers of notified STEC cases by federal state from 2015-2023 and relative change of case numbers in 2023 compared to 2022 and to the median of 2015-2019.

	Year of notification											
Federal State	2015	2016	2017	2018	2019	2020	2021	2022	2023	Median 2015-2019	fold-change 2023 in comparison to the median 2015-2019	fold-change 2023 in comparison to 2022
Baden-Wuerttemberg	149	244	333	397	370	217	293	308	407	333	1.2	1.3
Bavaria	392	448	474	469	371	292	297	331	371	448	0.8	1.1
Berlin	123	152	158	135	121	107	88	80	120	135	0.9	1.5
Brandenburg	57	75	73	106	68	63	55	72	115	73	1.6	1.6
Bremen	4	3	10	16	9	7	5	11	19	9	2.1	1.7
Hamburg	49	68	68	90	100	58	58	48	86	68	1.3	1.8
Hesse	80	73	98	83	79	57	80	88	166	80	2.1	1.9
Lower Saxony	298	363	435	386	285	269	258	337	762	363	2.1	2.3
Mecklenburg Western Pomerania	78	66	72	51	57	96	63	46	92	66	1.4	2.0
North Rhine-Westphalia	340	464	521	503	526	337	498	619	1,475	503	2.9	2.4
Rhineland Palatinate	150	166	171	183	188	115	118	130	174	171	1.0	1.3
Saarland	12	12	10	16	17	10	10	14	22	12	1.8	1.6
Saxony	316	146	234	266	180	98	131	139	232	234	1.0	1.7
Saxony-Anhalt	98	121	172	134	105	73	93	71	143	121	1.2	2.0
Schleswig-Holstein	50	108	121	121	107	82	81	106	220	108	2.0	2.1
Thuringia	49	50	78	99	74	43	61	41	54	74	0.7	1.3
Total	2,245	2,559	3,028	3,055	2,657	1,924	2,189	2,441	4,458	2,657	1.7	1.8

Tables S2 a and b: Sex distribution of STEC notifications by year (a) in Lower Saxony and (b) in Germany between 2015 and 2023.

a

sex	Year of notification									
	**2015**	**2016**	**2017**	**2018**	**2019**	**2020**	**2021**	**2022**	**2023**	**Median 2015-2019**
male	144	176	228	194	142	111	119	149	364	176
female	154	186	205	189	142	157	136	187	398	186
diverse	0	0	0	0	0	0	0	0	0	0
not ascertained or not collected	0	1	2	3	1	1	3	1	0	1
total	298	363	435	386	285	269	258	337	762	363
	**Proportions**									
proportion male	48.3%	48.5%	52.4%	50.3%	49.8%	41.3%	46.1%	44.2%	47.8%	48.5%
proportion female	51.7%	51.2%	47.1%	49.0%	49.8%	58.4%	52.7%	55.5%	52.2%	51.2%
proportion diverse, not ascertainable or not collected	0.0%	0.3%	0.5%	0.8%	0.4%	0.4%	1.2%	0.3%	0.0%	0.3%

b

sex	Year of notification									
	**2015**	**2016**	**2017**	**2018**	**2019**	**2020**	**2021**	**2022**	**2023**	**Median 2015-2019**
male	1062	1193	1436	1523	1322	906	1044	1114	2077	1322
female	1181	1359	1584	1527	1331	1010	1135	1320	2373	1359
diverse	0	0	0	0	0	1	1	0	0	0
not ascertained or not collected	2	7	9	5	5	7	9	7	8	5
total	2245	2559	3029	3055	2658	1924	2189	2441	4458	2658
	**Proportions**									
proportion male	47.3%	46.6%	47.4%	49.9%	49.7%	47.1%	47.7%	45.6%	46.6%	49.7%
proportion female	52.6%	53.1%	52.3%	50.0%	50.1%	52.5%	51.9%	54.1%	53.2%	51.1%
proportion diverse, not ascertainable or not collected	0.1%	0.3%	0.3%	0.2%	0.2%	0.4%	0.4%	0.3%	0.2%	0.2%

Supplementary Figure S1

Survnet, 04.12.2024

STEC Lower Saxony, reference definition NO (without symptoms or without available information on the presence of symptoms)
Age group year of notification

	2015	2016	2017	2018	2019	2020	2021	2022	2023	Median 2015-2019
A00..04	7	12	12	10	7	2	6	13	22	10
A05..09	3	10	9	7	1	7	2	5	11	7
A10..14	3	4	5	4	2	3	3	3	2	4
A15..19	6	5	13	3	5	3	4	2	10	5
A20..24	18	18	25	16	2	7	8	9	8	18
A25..29	12	12	21	12	12	6	2	7	11	12
A30..39	14	23	30	17	10	8	8	12	21	17
A40..49	18	14	26	20	11	13	10	5	17	18
A50..59	16	22	21	17	8	9	9	10	17	17
A60..69	5	6	6	3	4	6	5	6	24	5
A70_	7	5	10	8	6	7	6	13	17	7
**Summe**	**109**	**131**	**178**	**117**	**68**	**71**	**63**	**85**	**160**	117

STEC Lower Saxony, reference definition either YES or NO (either with symptoms or without symptoms/without available information on the presence of symptoms)
Age group year of notification

	2015	2016	2017	2018	2019	2020	2021	2022	2023	Median 2015-2019
A00..04	34	77	68	77	56	44	75	80	115	68
A05..09	13	21	28	30	18	17	11	22	37	21
A10..14	13	10	15	19	10	16	8	5	31	13
A15..19	21	15	39	10	14	15	12	22	39	15
A20..24	31	28	37	31	11	18	14	29	37	31
A25..29	23	24	35	24	20	19	11	15	43	24
A30..39	27	46	60	44	29	22	21	28	76	44
A40..49	36	29	47	43	33	33	30	18	71	36
A50..59	38	46	39	40	26	33	25	32	84	39
A60..69	24	27	27	23	26	24	16	31	110	26
A70_	38	40	40	44	42	28	35	55	119	40
**Summe**	**298**	**363**	**435**	**385**	**285**	**269**	**258**	**337**	**762**	363

Proportions of reference definition NO amongst all notifications, 5 and 10 year interval, Lower Saxony

	2015	2016	2017	2018	2019	2020	2021	2022	2023	Median 2015-2019
A00..04	21%	16%	18%	13%	13%	5%	8%	16%	19%	16%
A05..09	23%	48%	32%	23%	6%	41%	18%	23%	30%	23%
A10..14	23%	40%	33%	21%	20%	19%	38%	60%	6%	23%
A15..19	29%	33%	33%	30%	36%	20%	33%	9%	26%	33%
A20..24	58%	64%	68%	52%	18%	39%	57%	31%	22%	58%
A25..29	52%	50%	60%	50%	60%	32%	18%	47%	26%	52%
A30..39	52%	50%	50%	39%	34%	36%	38%	43%	28%	50%
A40..49	50%	48%	55%	47%	33%	39%	33%	28%	24%	48%
A50..59	42%	48%	54%	43%	31%	27%	36%	31%	20%	43%
A60..69	21%	22%	22%	13%	15%	25%	31%	19%	22%	21%
A70_	18%	13%	25%	18%	14%	25%	17%	24%	14%	18%
total	37%	36%	41%	30%	24%	26%	24%	25%	21%	32%

Proportions of reference def NO amongst all notifications, 10-and 20 year interval, Lower Saxony

	2015	2016	2017	2018	2019	2020	2021	2022	2023	Median 2015-2019
0-9 years	21%	22%	22%	16%	11%	15%	9%	18%	22%	21%
10-19 years	26%	36%	33%	24%	29%	19%	35%	19%	17%	29%
20-39 years	54%	54%	58%	45%	40%	36%	39%	39%	26%	54%
40-59 years	46%	48%	55%	45%	32%	33%	35%	30%	22%	46%
60-69 years	21%	22%	22%	13%	15%	25%	31%	19%	22%	21%
70_	18%	13%	25%	18%	14%	25%	17%	24%	14%	18%
**total**	**37%**	**36%**	**41%**	**30%**	**24%**	**26%**	**24%**	**25%**	**21%**	**32%**

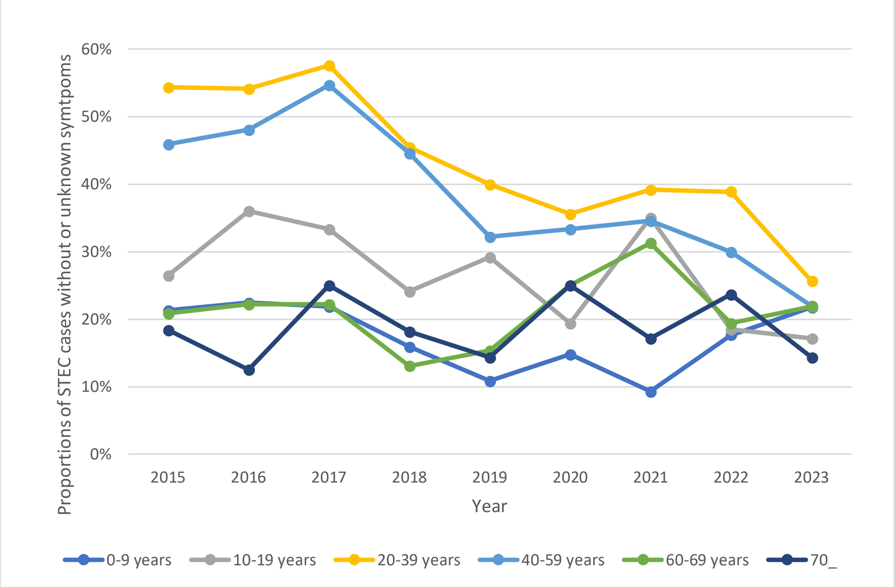

Figure 1S a: Proportions of STEC cases without symptoms or without available information on the presence of symptoms by year and age group, in Lower Saxony between 2015 and 2023.

STEC Germany, reference definition NO (without symptoms or without available information on the presence of symptoms)
Age group year of notification

	2015	2016	2017	2018	2019	2020	2021	2022	2023	Median 2015-2019
A00..04	80	87	115	128	135	76	114	133	148	115
A05..09	41	61	63	64	44	43	36	46	76	61
A10..14	23	26	35	28	39	23	23	23	46	28
A15..19	32	35	44	29	38	27	29	27	39	35
A20..24	60	54	76	67	43	33	41	33	39	60
A25..29	60	76	110	58	63	41	27	33	55	63
A30..39	94	113	183	141	122	87	73	74	125	122
A40..49	91	100	159	115	91	60	73	55	108	100
A50..59	57	87	103	89	81	46	47	58	109	87
A60..69	30	42	41	47	49	36	35	41	99	42
A70_	46	48	73	57	61	78	76	87	172	57

STEC Germany, reference definition either YES or NO (either with symptoms or without symptoms/without available information on the presence of symptoms)
Age group year of notification

	2015	2016	2017	2018	2019	2020	2021	2022	2023	Median 2015-2019
A00..04	570	606	706	843	731	504	739	667	863	706
A05..09	168	181	218	216	194	153	143	207	280	194
A10..14	108	111	124	125	111	89	81	90	193	111
A15..19	116	121	164	107	112	86	102	111	189	116
A20..24	136	135	165	165	113	82	86	104	179	136
A25..29	135	178	201	155	146	81	74	103	206	155
A30..39	214	271	354	312	277	183	170	197	432	277
A40..49	213	231	307	268	200	134	168	154	370	231
A50..59	180	251	257	282	249	164	167	225	441	251
A60..69	136	182	177	201	189	148	155	204	471	182
A70_	266	288	351	376	333	298	300	378	829	333

Proportions of reference definition NO amongst all notifications, 5 and 10-year interval, Germany

	2015	2016	2017	2018	2019	2020	2021	2022	2023	Median 2015-2019
A00..04	14%	14%	16%	15%	18%	15%	15%	20%	17%	15%
A05..09	24%	34%	29%	30%	23%	28%	25%	22%	27%	29%
A10..14	21%	23%	28%	22%	35%	26%	28%	26%	24%	23%
A15..19	28%	29%	27%	27%	34%	31%	28%	24%	21%	28%
A20..24	44%	40%	46%	41%	38%	40%	48%	32%	22%	41%
A25..29	44%	43%	55%	37%	43%	51%	36%	32%	27%	43%
A30..39	44%	42%	52%	45%	44%	48%	43%	38%	29%	44%
A40..49	43%	43%	52%	43%	46%	45%	43%	36%	29%	43%
A50..59	32%	35%	40%	32%	33%	28%	28%	26%	25%	33%
A60..69	22%	23%	23%	23%	26%	24%	23%	20%	21%	23%
A70_	17%	17%	21%	15%	18%	26%	25%	23%	21%	17%

Proportions of reference def NO amongst all notifications, 10-and 20 year interval, Germany

	2015	2016	2017	2018	2019	2020	2021	2022	2023	Median 2015-2019
0-9 years	16%	19%	19%	18%	19%	18%	17%	20%	20%	19%
10-19 years	25%	26%	27%	25%	35%	29%	28%	25%	22%	26%
20-39 years	44%	42%	51%	42%	43%	47%	43%	35%	27%	43%
40-59 years	38%	39%	46%	37%	38%	36%	36%	30%	27%	38%
60-69 years	22%	23%	23%	23%	26%	24%	23%	20%	21%	23%
70_	17%	17%	21%	15%	18%	26%	25%	23%	21%	17%

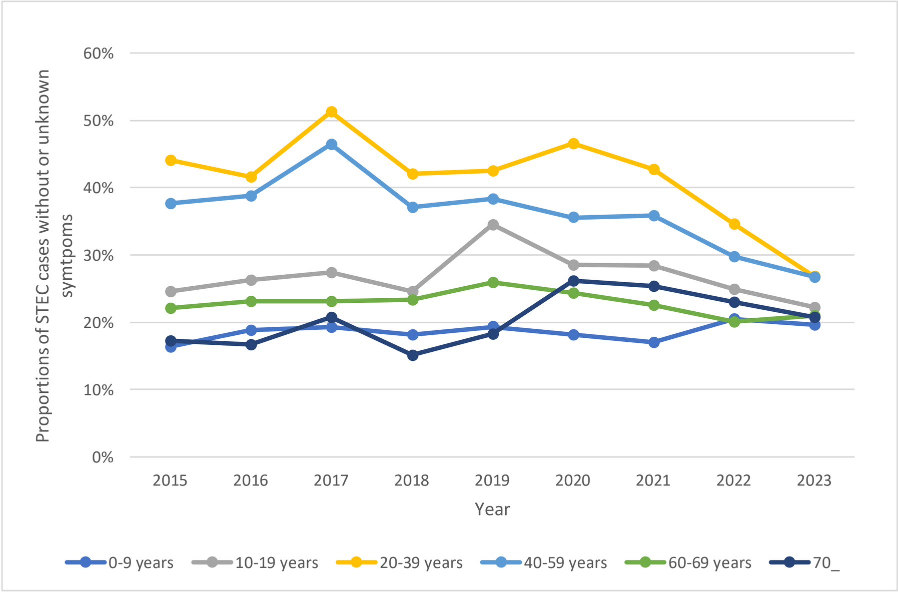

Figure 1S b: Proportions of STEC cases without symptoms or without available information on the presence of symptoms by year and age group, in Germany, between 2015 and 2023.
